# Case Report: ALK-positive histiocytosis presenting as an adrenal mass: a diagnostic trap due to unusual morphology

**DOI:** 10.3389/fonc.2026.1765507

**Published:** 2026-01-30

**Authors:** Wenjing Ma, Shenda Zhou, Jiali Lu, Haiming Wei, Yongta Huang

**Affiliations:** Department of Pathology, People’s Hospital of Guangxi Zhuang Autonomous Region (Guangxi Academy of Medical Sciences), Nanning, China

**Keywords:** adrenal gland, ALK, anaplasia, emperipolesis, histiocytosis

## Abstract

**Background:**

The differential diagnosis of an adrenal mass is critical for clinical management. We report a case that expanded the spectrum of a rare disease and present a novel diagnostic trap for both pathologists and clinicians.

**Methods:**

Histopathological, immunohistochemical (CD68, CD163, ALK), and molecular (FISH) analyses were performed on a right adrenal tumor from an 81-year-old male.

**Results:**

The tumor was diagnosed as ALK-positive histiocytosis. Uniquely, it presented as a primary adrenal mass with unusual morphology. The tumor cells showed marked anaplasia and emperipolesis. Apart from classic xanthogranuloma areas, the tumor exhibited hypercellular and hypocellular zones. Neoplastic cells in the hypercellular regions displayed anaplastic features, including a high nuclear-to-cytoplasmic ratio, prominent nucleoli, and observable mitotic figures. In contrast, neoplastic cells in the hypocellular areas were cytologically bland within a myxoid stroma. Neoplastic cells were positive for macrophage markers CD68, CD163 and ALK. Fluorescence *in situ* hybridization (FISH) demonstrated ALK gene rearrangements. The patient was disease-free after 8 months.

**Conclusion:**

To our knowledge, this is the first documentation of primary ALK-positive histiocytosis in the adrenal gland. The presence of anaplastic features poses a high risk of misdiagnosis as a sarcoma or carcinoma, potentially leading to overly aggressive therapy. Therefore, we advocate for the inclusion of this entity in the differential diagnosis of adrenal neoplasms and recommend routine ALK testing in similar challenging cases to guide precise management and avoid therapeutic errors.

## Introduction

Since its initial description in 2008, anaplastic lymphoma kinase (ALK)-positive histiocytosis has been recognized as a rare systemic neoplasm of histiocytic origin. The disease is characterized by the proliferation of bland, foamy histiocytes that exhibit ALK expression as a result of underlying ALK rearrangements (1). Currently, this disease was recognized as a distinct histiocytic neoplasm in both the 5th edition of the WHO classification and the International Consensus Classification (2, 3). The largest case series to date was published by Kemps et al. in 2022, comprising 39 patients (4). This series illustrated involvement of multiple systems, including the breast, skin, soft tissue, bone, lung, liver, and nervous system. Notably, despite this broad anatomical distribution, primary involvement of the adrenal gland has not been previously documented. Here, we report the first case of ALK-positive histiocytosis presenting as a primary right adrenal mass. The tumor exhibited unusual morphological features, including marked anaplasia and emperipolesis, which posed a significant diagnostic challenge. This study aims to delineate the clinicopathological characteristics of this novel presentation and thereby expand the known spectrum of this rare disease.

## Case report

The patient was an 81-year-old male whose right adrenal mass was an incidental finding on physical examination. The patient’s medical history was otherwise unremarkable. Computed tomography (CT) of the brain and chest revealed no obvious space-occupying lesions, while abdominal CT identified a 5-cm mass in the right adrenal gland. After the patient underwent surgical resection, it was observed that the mass was adherent to the liver. There was no radiographic or intraoperative evidence of involvement of the right renal parenchyma.

**Figure 1:**
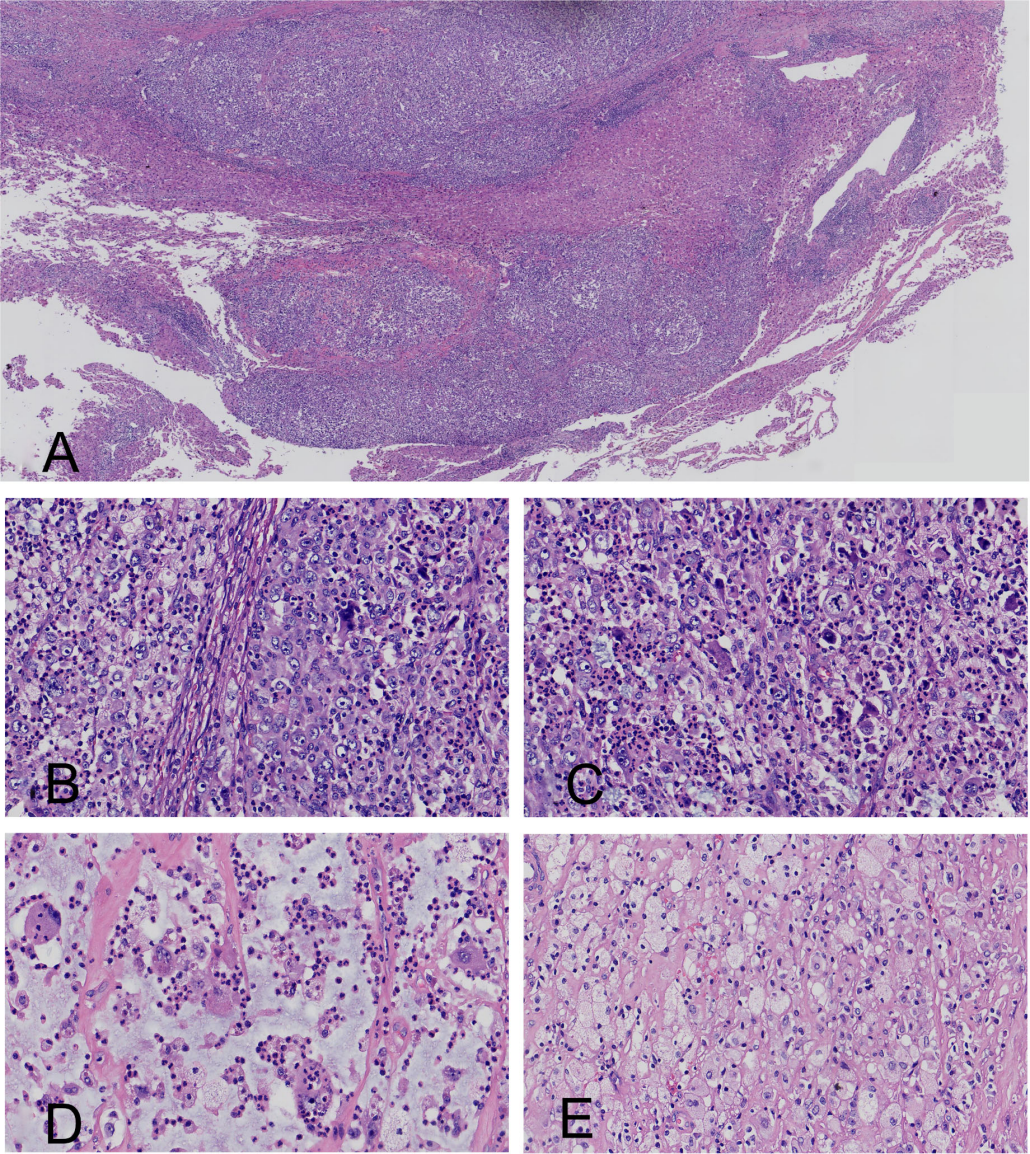
**(A)** Tumor invasion into the hepatic parenchyma. **(B)** High-power view showed a dense population of anaplastic tumor cells with irregular nuclear contours and prominent nucleoli. **(C)** Histologic features included mitotic figures and emperipolesis within the anaplastic tumor. **(D)** The hypocellular area showed cytologically bland tumor cells within a myxoid stroma. Emperipolesis was noted. **(E)** A classic xanthogranulomatous area. Emperipolesis was evident among the tumor cells.

The resected specimen was an irregular mass measuring 5.0 cm× 4.0 cm × 3.9 cm. Grossly, the tumor exhibited a partly gray-red and roughened external surface, with focal areas enveloped by adipose tissue. The cut surface was dark gray, firm, and lobulated. No identifiable normal adrenal tissue was observed. At low-power view, the tumor showed a diffuse proliferation of epithelioid cells, resulting in compression of the adrenal gland; focally, it infiltrated the adjacent adipose tissue and hepatic parenchyma (**Figure 1A**). At high magnification, the neoplastic cells demonstrated pleomorphic nuclei and conspicuous emperipolesis (lymphocytophagocytosis) of lymphocytes or neutrophils. The tumor is primarily composed of hypocellular areas, followed by classic xanthogranulomatous regions, with hypercellular areas being the least prevalent component. The cells within the hypercellular regions exhibit anaplastic features, including enlarged and multinucleated nuclei, irregular nuclear shapes, prominent nucleoli, and distinguishable mitotic figures (**Figures 1B, C**). Conversely, in the hypocellular areas, the cells exhibit abundant eosinophilic cytoplasm without significant atypical features, while the stroma demonstrates myxoid changes (**Figure 1D**). Focal areas displayed classic xantho-granulomatous morphology (**Figure 1E**). The tumor exhibited amphophilic cytoplasmic staining and demonstrated a diffuse infiltration of neutrophils. No tumor-associated necrosis was identified throughout the entire lesion. The residual adrenal tissue was severely compressed and largely effaced. This remaining tissue was histologically unremarkable, with no specific features like myelolipoma identified.

**Figure 2:**
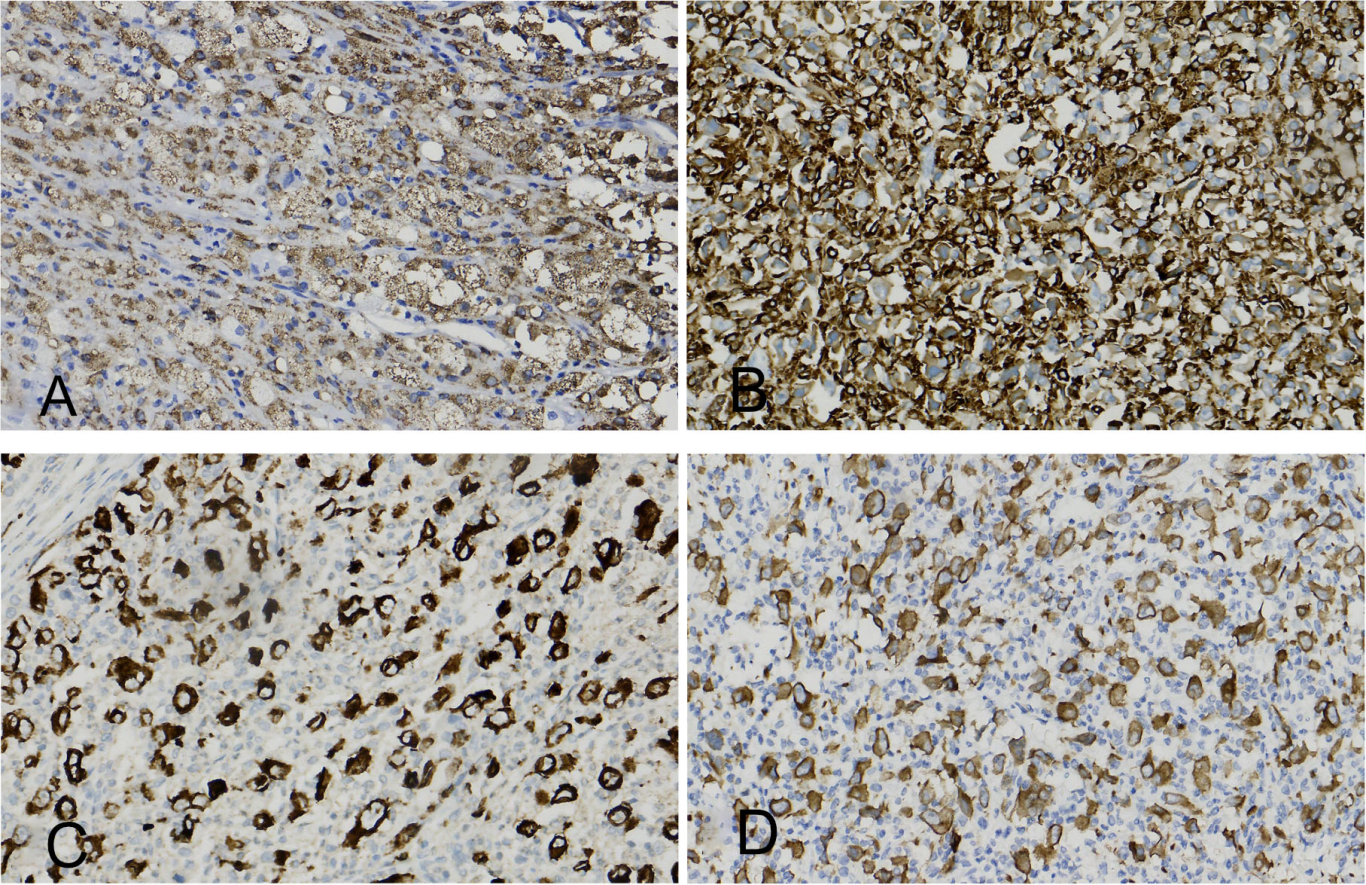
**(A)** The histiocyte marker CD68 is positive. **(B)** The histiocyte marker CD163 is positive. **(C)** The histiocyte marker ALK (Clone ID: D5F3) is positive (diffuse cytoplasmic positivity). **(D)** The histiocyte marker ALK (Clone ID: OTI1H7) is positive (diffuse cytoplasmic positivity).

**Figure 3:**
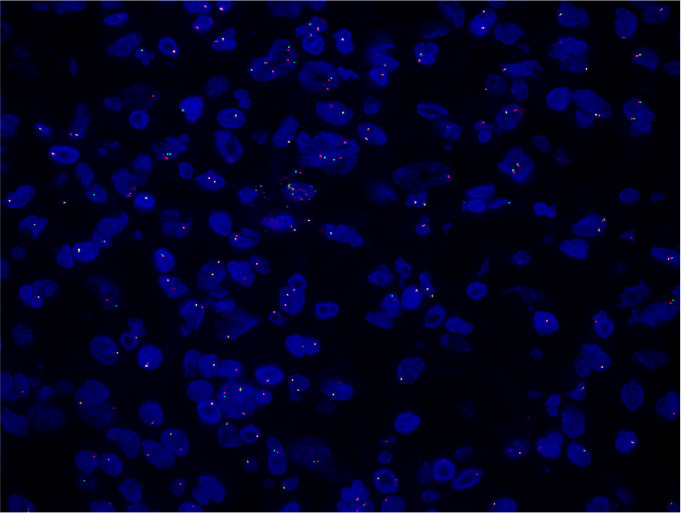
Fluorescence *In Situ* Hybridization testing was conducted on the adrenal mass, revealing ALK gene rearrangement.

Immunohistochemical staining revealed positivity for CD68, CD163, ALK (Clone ID: D5F3)and ALK (Clone ID: OTI1H7) (**Figures 2A-D**) and negativity for CK (AE1/AE3), S-100, HMB-45, Melan-A, EMA, HepPar-1, SF-1, CD1a, LCA, CD30, CD3, CD20, Langerin, SMA and desmin. A diffuse cytoplasmic staining pattern for ALK was observed in the tumor cells with both antibodies. The Ki-67 proliferation index in hypercellular areas was approximately 10%. Fluorescence *in situ* hybridization (FISH) demonstrated ALK gene rearrangements (**Figure 3**). BRAF V600E mutation was not detected.

Based on integrated histopathological, immunohistochemical, and molecular findings, the diagnosis was ALK-positive histiocytosis. Following complete resection of the mass, the patient declined adjuvant therapy (e.g., ALK inhibitors) in favor of surveillance and remained recurrence-free at the 8-month postoperative follow-up.

## Discussion

ALK-positive histiocytosis is a unique clinicopathologic entity among histiocytic disorders that is caused by ALK gene alterations. It was first described in 2008 in three infant females who presented with pallor, hepatosplenomegaly, and varying degrees of anemia (1). The same research group later reported an additional seven cases. This expanded cohort included both young children with systemic diseases and older patients exhibiting localized symptoms. These observations were pivotal in broadening the defined clinicopathological spectrum of this condition (5). Since the initial description, sporadic cases have been reported across a wide age range, spanning from 7 hours after birth to 71 years of age (6, 7). Notably, the current case involves an 81-year-old patient, marking the oldest individual diagnosed with this condition to date. The distribution of involved sites in ALK-positive histiocytosis demonstrates a correlation with patient age. In cases involving infants, the disease primarily affects the liver and hematopoietic system. In contrast, older children and adult patients have more extensive tumor involvement. It affects not only the hematopoietic system but also the nervous, skeletal, pulmonary, hepatic, skin, lymphatic, male reproductive systems, and breast tissue (4, 8). This case had occurred in the adrenal gland, a site not previously reported in the literature, suggesting that the endocrine system may be involved in this disease. This case provides a new direction for the differential diagnosis of adrenal tumors in clinical practice.

Histologically, ALK-positive histiocytosis exhibits a wide array of morphological features that show subtle variations across different affected organs. Most cases demonstrate the classic microscopic characteristics of xanthogranuloma, defined by foamy histiocytes and Touton giant cells. These lesions are occasionally accompanied by epithelioid cells. The foamy histiocytes feature eosinophilic cytoplasm and oval nuclei. These nuclei may exhibit slight membrane folding or indentation. Mitotic figures are rarely observed. Two studies described a morphologically variant form of ALK-positive histiocytosis. The tumor cells exhibited eosinophilic cytoplasm, indistinct cell borders, and nuclei that were oval to elongated and spindle- shaped (8, 9). However, the current case differed from previous reports due to unusual morphology. Tumor cells in this entity typically exhibit pleomorphic nuclei and conspicuous emperipolesis of lymphocytes or neutrophils. Of greater significance, the tumor cells in the dense regions exhibited pronounced anaplasia. Although these cells constitute only a relatively small proportion of the overall tumor, their marked anaplastic features were notable. The key characteristics of these cells included nuclear enlargement, a high nuclear-cytoplasmic ratio, irregular nuclear morphologies, prominent nucleoli, and distinguishable mitotic figures. These atypical tumor cells posed a significant diagnostic challenge, as they could easily be misdiagnosed as highly aggressive tumors, such as histocytic sarcoma. At the same time, the observation of histiocytes with engulfed lymphocytes also raised the possibility of Rosai-Dorfman disease. In conclusion, these atypical morphological findings significantly hindered the establishment of a correct pathological assessment. Consequently, immunohistochemical and FISH analyses proved critical and were essential for rendering a conclusive diagnosis in this case. The primary clinical significance of this case lies in its value as a diagnostic pitfall. In the context of an adrenal mass, the radiological and clinical presentation is non-specific. The morphological features observed, including the anaplastic areas, can easily lead to a misdiagnosis of more common entities, such as adrenal cortical carcinoma, metastatic carcinoma, or even a sarcoma. In this context, a benign or low-grade process like histiocytosis is virtually excluded from consideration. An erroneous diagnosis would have led to overly aggressive surgery (e.g., en bloc resection) or inappropriate chemotherapy. Therefore, this case mandates the inclusion of ALK-positive histiocytosis in the differential diagnosis of adrenal neoplasms, prompting the use of a simple immunohistochemical stain (ALK) that can prevent a major diagnostic error and guide appropriate management.

According to existing literature, tumor cell infiltration into the brain parenchyma has been documented in a 51-year-old patient with ALK-positive histiocytosis (9) Furthermore, other researchers have instances of vascular invasion by tumor cells in affected individuals (10). In this case, the tumor is situated within the adrenal gland parenchyma, penetrating the adrenal capsule and subsequently infiltrating the liver parenchyma. These findings suggest that ALK-positive histiocytosis may exhibit potential invasiveness. However, their prognostic significance remains uncertain and warrants further study. The presence of focal anaplastic features within our case raises intriguing questions about the biological spectrum of this disease. This suggests that ALK-positive histiocytosis may not always follow an indolent course and could have the potential for more aggressive clinical behavior. This finding calls for caution among clinicians and indicates that patients with similar histologic features may require closer clinical follow-up to monitor for potential recurrence or progression, even after complete resection.

Kemps et al. summarized 39 cases of ALK-positive histiocytosis, in which the KIF5B-ALK gene fusion was identified in approximately 70% of tumor tissue samples (4). In these cases, ALK immunohistochemical staining primarily displayed diffuse cytoplasmic positivity. Similarly, in the present case, the tumor tissue exhibited diffuse cytoplasmic staining for ALK on immunohistochemistry, suggesting a possible association with the KIF5B-ALK fusion. However, next-generation sequencing could not be performed due to limitations in funding.

This case necessitates a clear differentiation from histiocytic sarcoma. Histiocytic sarcoma is characterized by an aggressive clinical course, often marked by frequent recurrences and metastases (11). The tumor cells exhibit significant cellular heterogeneity, characterized by focal necrosis and pathological mitotic figures, along with a high Ki-67 proliferation index. Furthermore, the tumor is associated with genetic alterations in the MAPK pathway, with BRAF gene mutations present in 57% to 87% of cases (12–15). Notably, histiocytic sarcoma is consistently devoid of ALK rearrangements. In contrast, while focal tumor cells in our case displayed atypia accompanied by nuclear pleomorphism, definitive evidence of geographic necrosis or pathological mitotic figures was not observed. The Ki-67 proliferation index remained low; moreover, immunohistochemical analysis revealed strong ALK expression, and molecular testing confirmed the presence of ALK rearrangement without any detectable BRAF mutation. Collectively, this evidence supports the diagnosis of ALK-positive histiocytosis. Furthermore, during our 8-month follow-up period, the patient has remained free from recurrence, further substantiating our diagnosis. The tumor cells exhibited prominent emperipolesis, necessitating differential diagnosis from Rosai-Dorfman disease. Immunohistochemically, Rosai-Dorfman disease histiocytes are typically positive for S100, CD68, and CD163 (16). In this case, the tumor cells were negative for S-100 on immunohistochemistry, thus ruling out the diagnosis of Rosai-Dorfman disease.

This study has limitations. ALK-positive histiocytosis includes both multisystem and localized forms. Our assessment for systemic involvement was not comprehensive. Although computed tomography (CT) of the brain, chest, and abdomen revealed no diseases beyond the adrenal gland, neither whole - body imaging (e.g., PET - CT) nor bone marrow examination was conducted. Consequently, although our findings suggest an isolated adrenal lesion, systemic involvement cannot be entirely excluded. This underscores a common diagnostic dilemma when evaluating a seemingly solitary mass of this rare entity.

## Conclusion

In summary, this is the first reported case of ALK-positive histiocytosis arising in the right adrenal gland. The patient was 81 years old, which is a demographic feature distinctly uncommon for this disease. This case presented unusual morphological features, such as anaplastic cells, myxoid stromal changes, pleomorphic nuclei, and conspicuous emperipolesis, which posed a diagnostic challenge. It challenges the conventional view of ALK-positive histiocytosis as a uniformly low-grade morphological entity. The presence of anaplastic features introduces a significant diagnostic pitfall, which can lead to markedly different therapeutic decisions. Therefore, pathologists should consider this entity in the differential diagnosis of histiocytic neoplasms. It expands the differential diagnosis for adrenal masses, provides a rationale for routine ALK testing in histiocytic lesions at this site, and opens the door to potential targeted therapy, thereby bridging a morphological diagnosis directly to improved patient care.

## Data Availability

The original contributions presented in the study are included in the article/supplementary material. Further inquiries can be directed to the corresponding authors.
